# Regional lung function assessment using electrical impedance tomography in COPD, PRISm, and normal spirometry subjects: insights into early diagnostic potential

**DOI:** 10.1186/s12890-025-03668-z

**Published:** 2025-05-05

**Authors:** Jiayi Li, Zhanqi Zhao, Runze He, Yuhong Xie, Zhihao Xu, Chunwei Ni, Ting Jiang, Huiqing Ge

**Affiliations:** 1 Zhejiang University, School of Medicine, Hangzhou, China; 2 School of Biomedical Engineering, Guangzhou Medical University, Guangzhou, China; 3 Institute of Technical Medicine, Furtwangen University, Villingen-Schwenningen, Germany; 4 Department of Respiratory and Critical Care Medicine, the Fourth Affiliated Hospital of School of Medicine, Zhejiang University, Yiwu, China; 5 Department of Respiratory Therapy, Sir Run Run Shaw Hospital, Zhejiang University, School of Medicine, Hangzhou, China; 6 Taizhou Hospital of Integrated Traditional Chinese and Western Medicine, Taizhou, China; 7 Department of Respiratory and Critical Care Medicine, Sir Run Run Shaw Hospital, Zhejiang University, School of Medicine, Hangzhou, China

**Keywords:** Electrical impedance tomography, Preserved ratio impaired spirometry, Chronic obstructive pulmonary disease, Spatial–temporal heterogeneity, Regional lung function impairment

## Abstract

**Purpose:**

This study utilizes electrical impedance tomography (EIT) to explore spatial–temporal heterogeneity in regional lung function among patients with chronic obstructive lung disease (COPD), preserved ratio impaired spirometry (PRISm), and those with normal lung function.

**Methods:**

Subjects who had pulmonary function test at Sir Run Run Shaw Hospital from 28 December 2023 to 30 March 2024 were screened. Regional lung functions were accessed with EIT regarding spatial distribution, abnormal area size, and expiratory time. The correlations between smoking index, SGRQ score, and EIT-related parameters were also evaluated.

**Results:**

A total of 194 patients were screened and 161 patients were included (56 COPD, 21 PRISm, and 84 normal). Spatial distribution of regional FEV1EIT (*P* < 0.001), FVCEIT (*P* = 0.025), FEV1/FVCEIT (*P* < 0.001), MMEFEIT (*P* = 0.012), T-75EIT (*P* < 0.001), and FIVCEIT (*P* = 0.020) showed significant differences among the three groups. The percentage of abnormal FEV1/FVCEIT areas detected via EIT was 83.40% (25–75% percentiles 52.29%-98.39%) in the COPD group, 25.46% (17.31%-41.31%) in the PRISm group, and 10.37% (3.34%-19.04%) in the normal group. The time constant map revealed that the patients with COPD exhibited the longest exhalation times. Elevated smoking index and SGRQ scores were associated with increased heterogeneity and larger areas of abnormal FEV1/FVCEIT.

**Conclusion:**

Through EIT-based pulmonary function assessment, it is possible to sensitively identify the spatio-temporal heterogeneity in COPD and PRISm patients. Regional lung function impairments, particularly in PRISm patients with an FEV1/FVC ratio ≥ 0.7, were detected using EIT, highlighting its potential for early COPD diagnosis.

**Supplementary Information:**

The online version contains supplementary material available at 10.1186/s12890-025-03668-z.

## Introduction

In 2023, the Global Initiative for chronic obstructive lung disease (GOLD) introduced PRISm, defined as a preserved ratio (FEV1/FVC ratio ≥ 0.7 after bronchodilation) with impaired spirometry (FEV1 < 80% of reference after bronchodilation) [1]. Recently, large cross-sectional and longitudinal studies have highlighted the prevalence of PRISm, which is estimated at 7.1–20.3% because of population heterogeneity [2–7]. The GOLD 2024 report states that PRISm represents an unstable phenotype that can transition to either normal or obstructed spirometry, with 20–30% of cases progressing to chronic obstructive pulmonary disease (COPD) [8]. A recent meta-analysis revealed significantly increased risks of all-cause, cardiovascular, and respiratory-related mortality in PRISm patients [9]. Currently, there are no established diagnostic or therapeutic guidelines for PRISm, but research emphasizes the importance of screening and identifying PRISm in clinical settings, with potential for follow-up and early intervention when necessary.

Pulmonary function testing (PFT), to be specific, forced vital capacity manuever is the gold standard for diagnosing PRISm, but it cannot be used to assess regional lung function. Imaging modalities such as X-rays and computed tomography (CT) can capture structural abnormalities in the lungs but have drawbacks such as radiation exposure and the inability to measure dynamic lung function directly. Electrical impedance tomography (EIT) is a rapidly developing imaging technology with advantages such as noninvasiveness, no radiation exposure, and simplicity of operation [10–12]. Combining EIT with PFT is a novel application in respiratory medicine that can be used to assess regional lung function and identify pathological spatial and temporal ventilation heterogeneity in patients with COPD [13, 14].

The aim of this study was to evaluate the characteristics of lung ventilation distribution among COPD, PRISm and normal lungs using EIT-based pulmonary function assessment, with the goal of determining whether EIT can be used to detect regional lung function impairment, particularly in PRISm patients.

## Materials and methods

This prospective observational study was approved by the local ethics committee (No. 2023–933-01). Informed consent forms were signed by the subjects, and the study was registered online (NCT06199258). We recruited patients with respiratory symptoms at Sir Run Run Shaw Hospital, all of whom were over 18 years old. The exclusion criteria included a history of pulmonary diseases other than chronic airway inflammation, prior pulmonary surgery or radiotherapy, inability to undergo or potential interference with EIT assessment, and inability to undergo or poor cooperation with PFT. Vulnerable populations were also excluded.

PFT was conducted concurrently with EIT examinations [15]. A total of 16 electrodes were attached on the chest circumference at the 4–5th intercostal space for EIT data collection, and one reference electrode was placed on the abdomen of each subject. All patients were studied in the sitting position using the Draeger device (PulmoVista 500). Patients were briefed on PFT procedures prior to EIT recordings. The FVC test was repeated 3–8 times, and the highest quality result was used for analysis. The impedance changes in each EIT electrode (especially the electrodes on both sides of the sternum and spine) were monitored to ensure that there were no significant changes or detachments in contact impedance during the entire test [16]. After the examination, each patient was asked to complete a basic information questionnaire, COPD Assessment Test (CAT), and St. George’s Respiratory Questionnaire (SGRQ).

The EIT measurement principle is based on repetitive alternating current applications between 16 adjacent pairs of electrodes. During each current application over one adjacent electrode pair, 13 remaining passive adjacent electrode pairs were used to measure the resulting voltages. After ventilation-related data were collected via EIT, regional lung function was analyzed via customized software (Matlab 2013a, Mathwork, MA, US). Following data reconstruction, each EIT image consisted of 32 × 32 pixels. Within the lung region pixels, the difference between the maximum relative impedance value (Z_max_) reached after full inspiration to total lung capacity and the minimum relative impedance value (Z_min_) reached subsequently after maximal expiration to residual volume was calculated. This difference reflects the forced vital capacity (FVC_EIT_) values at each pixel. Convert the impedance values to milliliters by comparing the sum of all pixel FVC_EIT_ values with the FVC measured by spirometry. Similarly, the 1-s forced expiratory volume (FEV1_EIT_) is calculated as the difference between ΔZ values one second after forced expiration in lung pixels and the lung volume at the start of expiration and the forced inspiratory vital capacity (FIVC_EIT_) is calculated as the difference between the ΔZ values corresponding to residual lung volume and total lung capacity after a maximal inspiration. Additionally, the global time points when the FVC reached 25% and 75% (MEF25 and MEF75) were determined. The mean flow rates were calculated at the pixel level and expressed as MMEF_EIT_. The pixel variation value representing the time required to exhale 75% of FVC is denoted as T-75, and it calculated the average required time. To describe the dispersion of fEIT images, global inhomogeneity (GI) [17] were computed for each type of fEIT. GI is calculated based on the difference between the average impedance change of the entire lung and the impedance change of each pixel generated during the imaging process, representing the dispersion of the ventilation distribution. A higher GI indicates greater heterogeneity in the lung [18]. Center of Ventilation (CoV) is an indicator for evaluating the changes in ventilation distribution in the vertical direction of the abdomen and back [19]. Pixels with an fEIT FEV1/FVC ratio less than 0.7 were counted as a percentage of total lung region pixels, represented as “abnormal%”. We calculated the lung size at maximum aspiration. With respect to temporal heterogeneity, a time constant (τ) map was created on the basis of calculations from FVC data [20]. For numerical statistics, the median (τ_med_) and interquartile range (τ_iqr_) of regional τ or the mean/variance in expiration time were used to represent the data. Furthermore, we examine the correlation between the smoking index (years of smoking × cigarettes smoked per day), symptom scores, and pulmonary ventilation heterogeneity. The primary endpoint was defined as EIT—related parameters, including the GI index, the proportion of regions with abnormal areas of FEV1/FVC, and the time constant.

Statistical analyses were performed using GraphPad Prism version 10.0 (GraphPad Software, San Diego, CA, USA). Quantitative indicators were compared by one-way ANOVA or Kruskal–Wallis test according to the data distribution, followed by Dunn’s multiple comparison test. Categorical indicators were compared by the chi-square test or Fisher’s exact test. For each group, the paired—samples t—test or the Wilcoxon signed—rank test was applied to analyze the data before and after the bronchodilation test.Correlation analysis was conducted with either Pearson or Spearman methods. A p-value of < 0.05 was considered statistically significant.

## Results

**Fig. 1:**
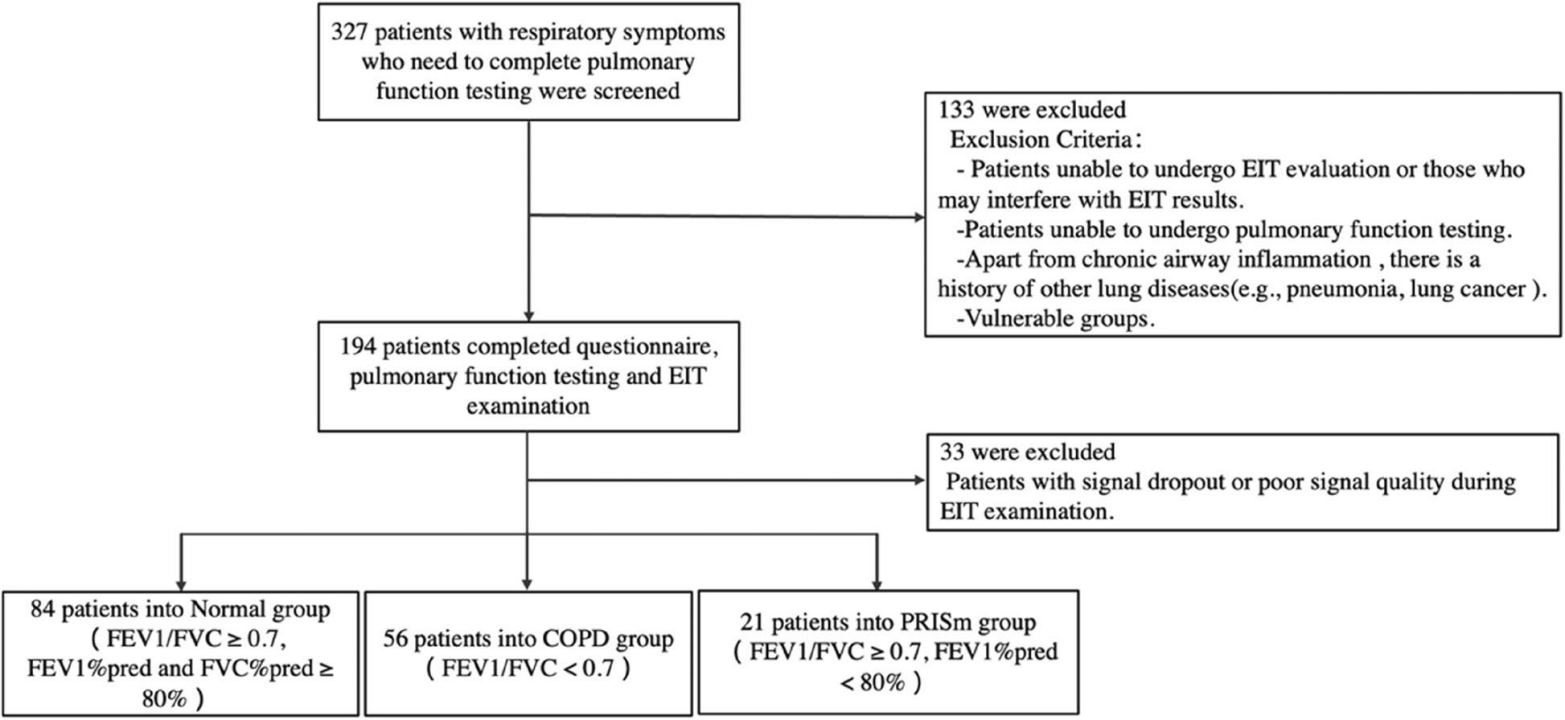
Enrolment and follow-up of the study participants

The enrolment and exclusion criteria are detailed in Fig. 1. From 28 December 2023 to 30 March 2024, a total of 194 patients successfully completed PFT, questionnaire surveys, and baseline analyses. In this study, 98 normal lung function individuals, 63 COPD patients, and 33 PRISm patients were included, with baseline characteristics reported in Table 1. The lung function process of all patients complies with quality control standards. Significant differences were observed across groups in terms of age, smoking history, history of bronchodilator use, and comorbidities, including hypertension and prior COPD diagnosis (*p* < 0.05). The COPD and PRISm groups demonstrating significantly higher symptom scores compared to the normal group.

**Table 1:** Study participants’ characteristic

Variables	Normal (*n* = 98)	COPD (*n* = 63)	PRISm (*n* = 33)	*P* value
Sex, n (%)				
Male	49 (50.0%)	53 (84.1%)	19 (57.6%)	.000
Female	49 (50.0%)	10 (15.9%)	14 (42.4%)	
Age (years)	54.72 ± 14.00	67.63 ± 9.79	59.15 ± 15.60	.000
BMI (kg/m2)	24.35 ± 3.39	24.03 ± 3.27	24.05 ± 3.91	.487
Smoking, n (%)				
Never	69 (70.4%)	22 (34.9%)	18 (54.5%)	.000
Former	9 (9.2%)	24 (38.1%)	8 (24.2%)	
Current	20 (20.4%)	17 (27.0%)	7 (21.2%)	
Previous diagnosis of COPD	2 (2.0%)	18 (28.6%)	3 (9.1%)	.000
Bronchodilators have been used, n (%)	15 (15.3%)	26 (41.3%)	5 (15.2%)	.000
Comorbidities, n (%)				
Hypertension	28 (28.6%)	25 (39.7%)	17 (51.5%)	.046
Diabetes	10 (10.2%)	7 (11.1%)	5 (15.1%)	.739
CHD	9 (9.2%)	9 (14.3%)	6 (18.1%)	.340
Tumor	1 (1.0%)	3 (4.8%)	1 (3.0%)	.338
Previous asthma	5 (5.1%)	2 (3.2%)	4 (12.1%)	.186
CAT score	11.16 ± 5.40	13.90 ± 6.53	14.42 ± 7.00	.010
SGRQ score	20.05 ± 10.43	29.95 ± 14.44	27.55 ± 15.24	.000

*BMI* body mass index, *COPD* chronic obstructive pulmonary disease, *CHD* coronary heart disease, *CAT* COPD Assessment Test, *SGRQ* St.George's Respiratory Questionnaire

**Fig. 2:**
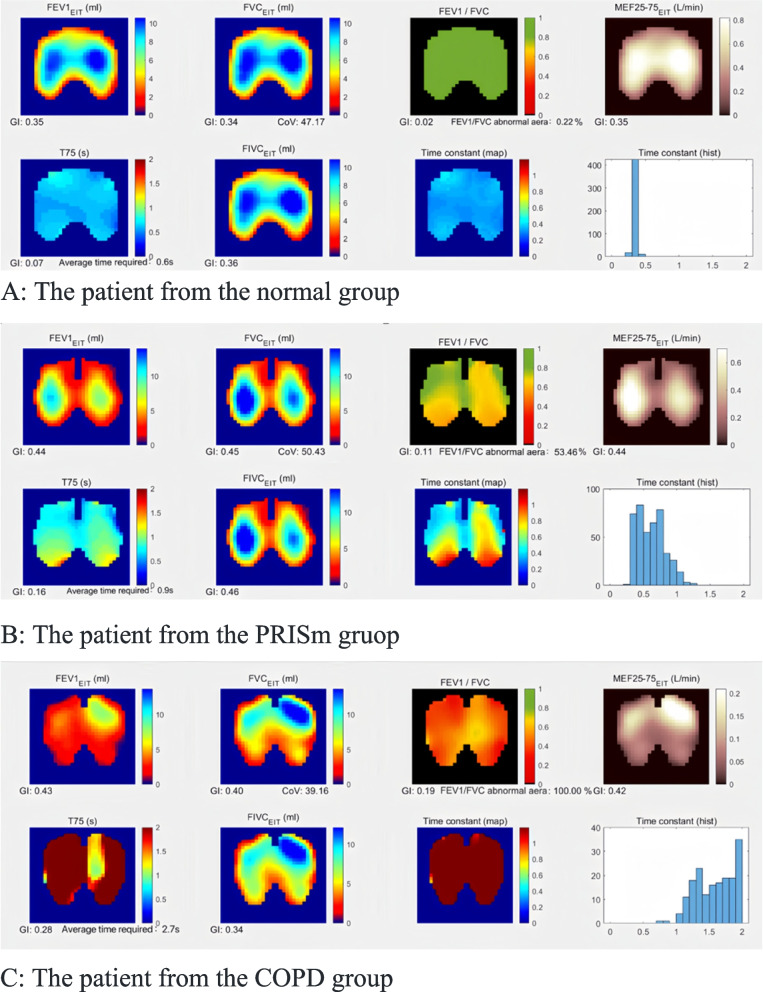
EIT images examples. C: The patient from the COPD group Global inhomogeneity (GI) of electrical impedance tomography derived regional pulmonary function measures of forced expiratory volume in 1 s (FEV1), forced vital capacity (FVC), FEV1/FVC, forced inspiration vital capacity (FIVC), middle expiratory flow rate at 25% to 75% of FVC (MEF25-75), time required to exhale 75% of FVC (T-75) in COPD, PRISm, Normal patients; FEV1/FVC abnormal aera: pixels with an fEIT FEV1/FVC ratio less than 0.7 of the total lung region pixels; CoV: center of ventilation; Averaged time required: the time required to exhale 75% of FVC; Time constant: as numerical statistics, the median (τmed) and interquartile range (τiqr) of regional time constants

Prior to conducting a quantitative analysis of ventilation heterogeneity, we excluded 33 patients with poor signal quality on the basis of EIT image inspection. The final cohort included 84 patients in the normal group, 21 patients in the PRISm group and 56 in the COPD group. The EIT imaging examples in Fig. 2 illustrates ventilation patterns in normal individuals compared to COPD and PRISm patients, with visible differences in lung dynamics.

Table 2 presents spirometry and EIT parameters pre- and post-bronchodilation across the groups. There are differences in the predicted values of FVC, FEV1, and FEV1/FVC ratio. After bronchodilation, statistical differences in GI of the regional FVC_EIT_ (*P* = 0.025), FEV1_EIT_ (*P* < 0.001), FIVC_EIT_(*P* = 0.020), FEV1/FVC_EIT_ (*P* < 0.001), MEF25-75_EIT_ (*P* = 0.012), and T-75_EIT_ (*P* < 0.001) were observed among the three groups. After multiple comparisons, the GI of FEV1_EIT_, FVC_EIT_, FIVC_EIT_, FEV1/FVC_EIT_, and T-75_EIT_ in the PRISm group was significantly higher than that in the normal group. According to the abnormal area defined by FEV1/FVC_EIT_, we found that the COPD group had the highest proportion of abnormal areas [83.40% (52.29%—98.39%)], followed by the PRISM group [25.46% (17.31%-41.31%)], and the normal group had the lowest [10.37% (3.34%-19.04%)], with statistically significant differences. The average required time, τ_iqr_, and τ_med_ in the COPD group were greater than those in the other two groups. The CoV shows a ventilation bias towards the ventral side in the COPD group compared to the other two groups. The normal group showed notable improvements in parameters such as GI of T75_EIT_, and τiqr after bronchodilation (*p* < 0.05), suggesting reduced lung resistance, whereas COPD and PRISm patients exhibited more limited improvements.

**Table 2:** EIT parameters and PFT parameters before and after the bronchodilation test

Variables	Normal (*n* = 84)			COPD(*n* = 56)			PRISm (*n* = 21)			*P*
	Before	After	*P*	Before	After	*P*	Before	After	*P*	After
GI FEV1 GI FVC CoV GI FEV1/FVC FEV1/FVC abnormal area GI-MEF25-75 GI-T75 Average time required GI-FIVC Lung Size τmed τiqr FVC% pred FEV1%pred FEV1/FVC	0.39 (0.36–0.41) 0.39 (0.36–0.42) 50.38 ± 4.09 0.11(0.08–0.18) 12.75 (5.58–22.23) 0.39 (0.37–0.41) 0.15 (0.10–0.21) 0.75 (0.65–0.80) 0.38 (0.36–0.41) 396.76 ± 56.36 0.48 (0.38–0.58) 0.16 (0.09–0.28) 98.56 ± 11.61 95.63 ± 10.72 79.73 ± 6.21	0.39 (0.36–0.41) 0.39 (0.36–0.43) 49.93 ± 3.71 0.10(0.07–0.17) 10.37 (3.34–19.04) 0.39 (0.36–0.41) 0.12 (0.09–0.17) 0.70 (0.60–0.80) 0.38 (0.35–0.42) 409.52 ± 53.03 0.42 (0.35–0.55) 0.12 (0.05–0.22) 98.35 ± 11.55 97.62 ± 10.39 81.70 ± 5.92	.840 .757 .068 .380 .031 .889 .007 .000 .690 .003 .000 .003 .634 .000 .000	0.42 (0.38–0.46) 0.39 (0.36–0.43) 47.75 ± 4.45 0.17 (0.13–0.23) 82.93 (66.81–95.48) 0.41 (0.38–0.46) 0.29 (0.22–0.37) 1.85 (1.30–2.58) 0.40 (0.35–0.43) 425.43 ± 55.48 1.33 (1.06–1.83) 0.55 (0.40–0.69) 79.16 ± 16.32 58.08 ± 17.62 56.98 ± 9.65	0.41 (0.38–0.45) 0.39 (0.37–0.42) 47.95 ± 4.40 0.17 (0.12–0.21) 83.40 (52.29–98.39) 0.40 (0.37–0.44) 0.28 (0.21–0.36) 1.70 (1.20–2.36) 0.39 (0.36–0.43) 436.39 ± 55.67 1.20 (0.87–1.67) 0.50 (0.37–0.60) 82.35 ± 16.92 61.56 ± 18.33 57.72 ± 9.82	.519 .499 .552 .522 .082 .468 .974 .012 .708 .110 .000 .150 .000 .000 .175	0.40 (0.37–0.46) 0.39 (0.36–0.46) 50.60 ± 3.39 0.14 (0.10–0.19) 24.19 (18.78–43.66) 0.40 (0.36–0.44) 0.18 (0.15–0.24) 0.90 (0.70–1.00) 0.39 (0.36–0.46) 396.19 ± 41.37 0.59 (0.41–0.70) 0.25 (0.13–0.32) 75.60 ± 7.44 69.50 ± 7.69 73.68 ± 6.03	0.43 (0.38–0.46) 0.42 (0.38–0.45) 51.03 ± 3.72 0.14 (0.12–0.29) 25.46 (17.31–41.31) 0.42 (0.38–0.46) 0.20 (0.15–0.24) 0.80 (0.60–0.90) 0.42 (0.39–0.46) 417.33 ± 56.16 0.53 (0.39–0.67) 0.25 (0.14–0.36) 79.04 ± 8.99 73.79 ± 5.42 76.15 ± 4.41	.619 .193 .408 .096 .633 .268 .615 .007 .248 .016 .109 .176 .062 .005 .007	.000 .025 .003 .000 .000 .012 .000 .000 .020 .022 .000 .000 .000 .000 .000

The *p*—value following each group indicates whether there is a statistically significant difference before and after the bronchodilation test for that group. “P after” refers to whether there is a statistically significant difference among the three groups after the bronchodilation test. *EIT* electrical impedance tomography, *PFT* pulmonary fucnction tests, *GI* global inhomogeneity, *FEV1* forced expiratory volume in 1 s, *FVC* forced vital capacity, *MEF* maximal expiratory flow, *FIVC,* forced inspiration vital capacity

**Fig. 3:**
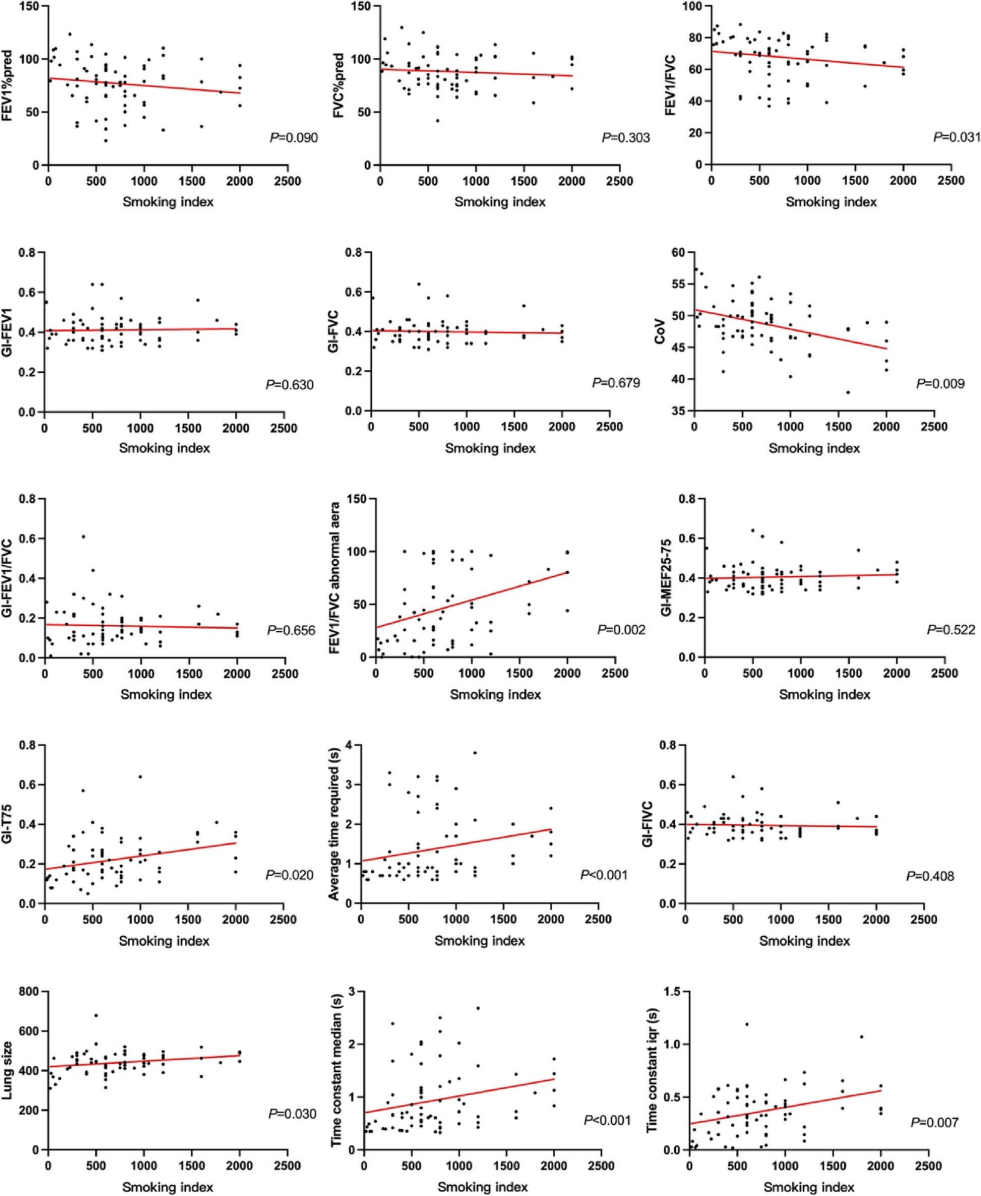
Smoking index correlation analysis. EIT: electrical impedance tomography. PFT: pulmonary function tests. GI: global inhomogeneity. FEV1: forced expiratory volume in 1 s; FVC: forced vital capacity. MEF: maximal expiratory flow. FIVC: forced inspiration vital capacity

**Fig. 4:**
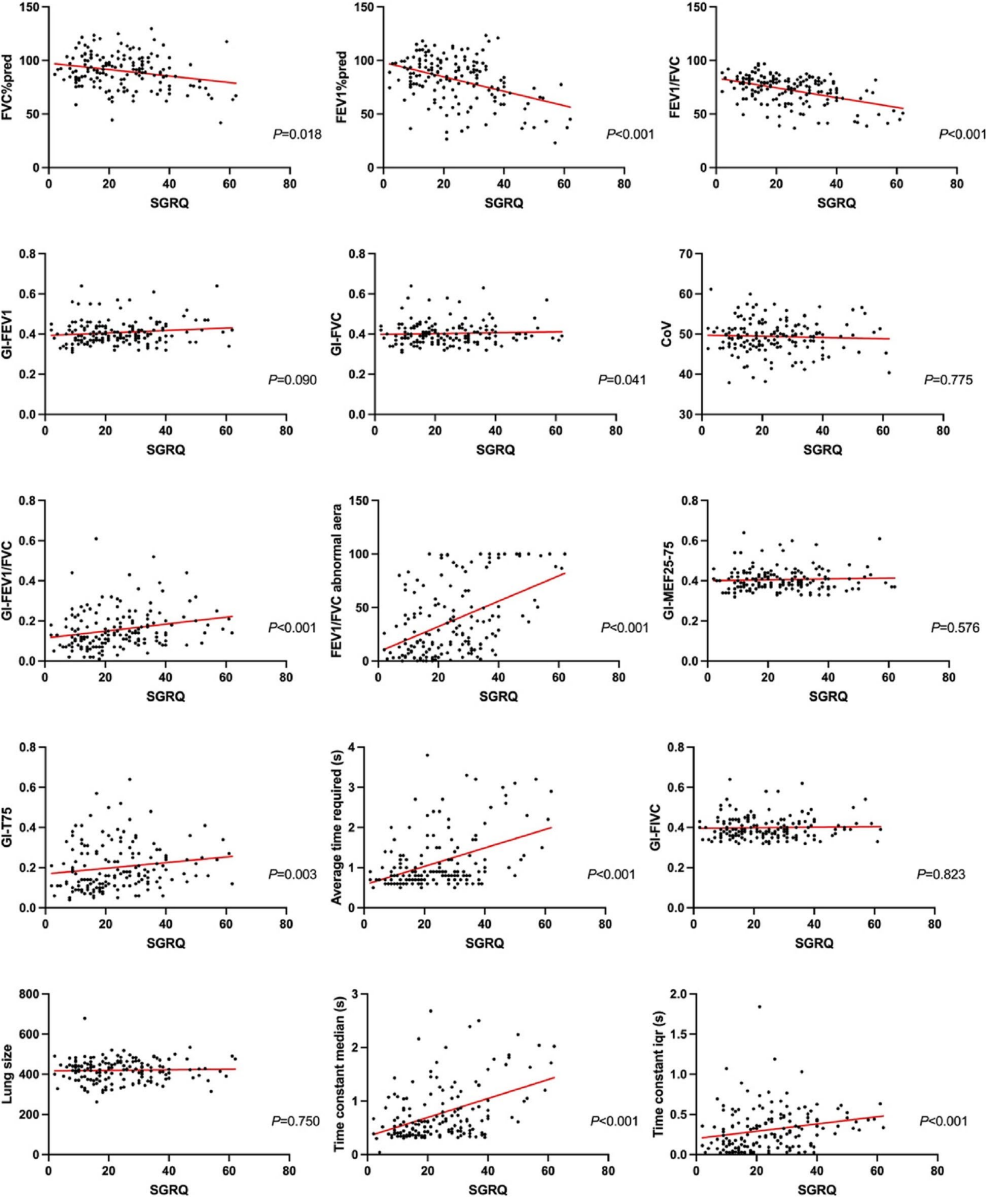
SGRQ score correlation analysis. EIT: electrical impedance tomography. PFT: pulmonary function tests. GI: global inhomogeneity. FEV1: forced expiratory volume in 1 s; FVC: forced vital capacity. MEF: maximal expiratory flow. FIVC: forced inspiration vital capacity

Finally, Fig. 3 presents a correlation analysis between smoking index and lung function metrics, showing significant associations noted for FEV1/FVC_EIT_ abnormal area, GI of T75_EIT_, CoV, average time required, τmed and τiqr. Figure 4 further correlates SGRQ scores with lung function, revealing a correlation with GI of FEV1/FVC_EIT_ and T-75_EIT_, the abnormal areas of FEV1/FVC_EIT_, average time required, τmed, and τiqr.

## Discussion

In the present study, spatial and temporal heterogeneity in regional lung function was found in patients with COPD and PRISm compared to that in normal lungs subjects. The baseline analysis revealed that among the population diagnosed with COPD, only 28.6% had a prior formal diagnosis, whereas 41.3% reported a history of bronchodilator use. The COPD patient population is predominantly male, older, and exhibits a high prevalence of both former and current smoking history, which aligns with the findings of the current review [21]. We also found that the hypertension prevalence, CAT score, and SGRQ score were significantly greater in the PRISm group than in the normal group, and the symptom score was similar to that of COPD patients. This finding is consistent with findings in previous studies that showed that patients with PRISm had significantly increased respiratory symptoms and cardiovascular disease burden [22]. Currently, there is no research elucidating the causal pathways between PRISm and comorbidities, potentially due to environmental or genetic factors [23].

EIT has been extensively utilized in experimental and clinical research [24–26], although its application during PFT is relatively infrequent. In our study, patients were seated with the EIT electrode plane oriented perpendicular to the gravity vector [27]. We quantified the spatial and temporal heterogeneity of lung ventilation via EIT by analyzing the ventilation distribution on the basis of all pixel values derived from EIT measurements of lung volume. Research combining EIT with PFT has been conducted to investigate the spatial and temporal heterogeneity of ventilation in young individuals, elderly individuals, and patients with COPD [13], and the results indicated that EIT can provide supplementary information during PFT and help with identifying pathological spatial and temporal heterogeneity in regional lung function. In the present study, we utilized the GI index, as recommended for clinical practice [28], to assess spatial and temporal heterogeneity.

The results of our study showed that EIT-based lung function parameters can be used to assess differences in spatial and temporal ventilation distribution between COPD patients, PRISm patients, and those with normal lung function, with higher levels of ventilation heterogeneity observed in COPD and PRISm patients (Table 2). Compared to pulmonary function tests, EIT images can locate regions with abnormal volume decreases and prolonged time anomalies, allowing for early identification of regional lung dysfunction and injury. Previous study has suggested that bronchial dilation therapy can influence regional EIT-derived lung function measurements. Specifically, in asthmatic patients, the regional ventilation distribution tends to improve following bronchodilator use [29]. Therefore, we assessed lung function after bronchodilation to minimize the impact of airway hyperresponsiveness on the patients. FVC, FIVC, and FEV1 are mainly used as volume indicators [30], and the EIT images of these indicators show the characteristics of volume changes in different regions. The GI values of FEV1_EIT_, FVC_EIT_, and FIVC_EIT_ offered a consolidated view of the spatial ventilation distribution across the chest region. Higher GI values correspond to increased inhomogeneity in the pulmonary ventilation. Overall, the pulmonary spatial heterogeneity of patients with COPD and PRISm is higher than that of the normal lung function. FEV1/FVC ratio, MMEF25-75% and T-75 are mainly used to assess airflow obstruction indicators [30]. The proportion of pixels with an FEV1/FVC_EIT_ ratio less than 0.7, relative to the total number of pixels within the lung region, was highest in the COPD group, and the proportion of abnormal areas in the PRISm group was higher than that in the normal group, exhibiting statistically significant differences.The GI vaules of FEV1/FVC_EIT_ evaluates the heterogeneity of the decline in the forced expiratory volume in one second. The pathogenesis of COPD is rooted in the innate and adaptive immune responses triggered by the inhalation of toxic particles and gases. As the disease advances, several pathological changes occur, including structural obstruction and alterations in peripheral airways, airway remodeling due to airway stenosis and peritubular fibrosis, destruction of lung parenchyma, development of emphysema, and modifications in pulmonary vasculature. These changes collectively contribute to uneven lung ventilation and a decreased FEV1/FVC ratio [31–33]. The pathology of PRISm remains unclear. A multicenter prospective study revealed that inflammatory markers are associated with a decline in FEV1 in no pulmonary disease populations [34]. A cross-sectional study revealed that PRISm is associated with small airway dysfunction and reduced total lung capacity [35]. EIT-based regional lung function measurements enable the sensitive detection of early regional lung function impairment, especially in PRISm patients, indicating changes in regional FEV1/FVC_EIT_ < 0.7. This reduction in the one-second rate may be related to physiological abnormalities and structural lung pathology in this region, as well as inflammation, air trapping, hyperinflation, and reduced lung diffusing capacity. Inflammation and abnormalities in airway structure and function are important not only for the occurrence and development of COPD but also for similar changes in pre-COPD and PRISm patients [36]. In our study, the average time required to exhale 75% of the gas was 0.70 (0.60–0.80) s in the normal group, 0.80 (0.60–0.90) s in the PRISm group, and 1.70 (1.20–2.36) s in the COPD group. Pulmonary function tests in COPD patients shows prolonged expiratory time on the time-volume curve, with failure to reach the expiratory plateau or reaching the plateau in more than 6 s [37, 38]. The GI of T-75_EIT_ value shows the temporal heterogeneity of regional lung function. Although there was no significant difference in average time between the PRISm and normal groups, the temporal heterogeneity was greater in the PRISm group. This suggests that patients in the PRISm group have lung regions with abnormally prolonged expiratory time. The time constant map reflects the rate of lung inflation or deflation; a larger value indicates slower inflation or deflation, while a smaller value indicates faster inflation or deflation [39, 40]. In our study, we calculated the FVC_EIT_ time constant map, which mainly provides an objective assessment of lung compliance, airway resistance, respiratory muscle power, and endurance [20]. The τiqr and τmed in the COPD group are significantly longer than those in the other two groups. The longer the time, the greater the likelihood of lung injury. The CoV primarily reflects the characteristics of the ventilation distribution in the dorsal–ventral axis. Dorsal region collapse shifts the CoV upwards (< 50%), while overinflation shifts the CoV downwards (> 50%) [28, 41]. Patients with COPD showed a greater deviation from the 50% value compared to the other two groups, indicating a greater degree of lung injury.

Bronchodilators can reduce airway resistance and are manifested in EIT as a shorter time constant. We compared the data of patients before and after bronchodilation and detected an improvement in the post-bronchodilation temporal heterogeneity of the lungs in patients (Table 2). A previous study integrated the characteristics of COPD patients with positive and negative bronchodilation responses via the combination of PFT and EIT [42]. Both groups exhibited an inhomogeneous ventilation distribution. Significant improvements were observed in the spatial distribution of the pixel FEV1 and tidal volume, as well as in the temporal distribution, among positive responders. Owing to the paucity of patients exhibiting positive bronchodilator responses, further comparative analysis was precluded within the current study. However, we intend to expand our cohort in subsequent studies to thoroughly investigate the distinctions between positive and negative responders.

Smoking can lead to an increase in pulmonary ventilation heterogeneity. EIT is capable of evaluating the spatial and temporal distribution of lung function in adults without pulmonary disease and identifies an increased heterogeneity in former and current smokers compared to nonsmokers [43]. In COPD and PRISm patients, smoking is identified as a pivotal environmental risk factor [7, 44]. Previous studies have demonstrated that smoking is associated with a decline in lung function [45, 46]. We found that a higher smoking index correlates with a greater decrease in FEV1/FVC. Similarly, in the regional lung function evaluated by EIT, the proportion of areas with abnormal FEV1/FVC also increases (Fig. 3). This further indicates that EIT can provide additional information, particularly in assessing spatial and temporal characteristics.

The more severe the respiratory symptoms, the higher the pulmonary ventilation heterogeneity may be. We found a strong correlation between symptom scores and the partial temporal and spatial heterogeneity indices provided by EIT (Fig. 4). On the basis of the results of traditional PFT, some patients present significant symptoms, but their FEV1/FVC ratio is greater than 0.7. This discrepancy may be because traditional spirometry captures changes in airflow in the central airways but lacks spatial information. Regional lung function impairment may be masked by other normal regions. Therefore, it is common to overlook these types of patients in clinical practice. These findings demonstrate that combining EIT with PFT can provide additional information for clinical diagnosis and treatment.

The authors of one study proposed the use of quantitative HRCT imaging to evaluate PRISm characteristics, revealing significant differences between PRISm and small airway and vascular abnormalities in patients with normal lung function. CT appears to be more sensitive than PFT parameters for detecting early-stage COPD [47]. However, the primary concern is exposure to ionizing radiation, and a comprehensive evaluation requires advanced biphasic CT scans, which include both deep inspiration and expiration phases, thereby increasing the radiation dose. This significantly limits the clinical applicability of this approach. Currently, multiple studies have utilized quantitative dynamic digital radiography (DDR) to evaluate the pulmonary function of patients with COPD. The studies have confirmed the association of lung signal intensity changes during forced breathing with pulmonary function and disease severity. However, DDR lacks lateral information, which may limit its comprehensive assessment of the lungs [48, 49]. ^12^⁹Xe MRI can characterize pulmonary ventilation. Models trained on COPD patients showed high accuracy but didn’t study those with different characteristics [50]. EIT is characterized by its noninvasive, simple, and repeatable nature. Multiple clinical studies have demonstrated that EIT exhibits good concordance and correlation with CT and PFT, effectively reflecting pulmonary gas distribution under different ventilation conditions [51, 52]. Several clinical studies have reported the application of regional lung function assessment via EIT in patients with chronic lung diseases [53, 54]. Research has shown that this technology can effectively differentiate between patients and healthy individuals. By providing additional regional lung function information beyond that provided by PFT, EIT facilitates comprehensive, personalized treatment.

Our study has several limitations. First, the EIT measurements were generally obtained with patients in a seated position, which might result in suboptimal electrode contact on the lateral aspects of the spine and sternum. At present, we rely primarily on adhesive tape for electrode fixation; however, future advancements may offer more effective solutions to address this limitation. Another limitation of our study is the absence of long-term follow-up. Since substantial changes in pulmonary function are unlikely to occur within a short time frame, future research should involve a larger cohort and extended follow-up periods to evaluate the clinical relevance of EIT in assessing regional lung function. Finally, our study did not compare EIT with other imaging modalities such as HRCT.

## Conclusion

In conclusion, we first showed that EIT—based regional lung function can assess outpatients’ lung status with respiratory symptoms, sensitively distinguishing heterogeneity differences among COPD, PRISm patients and those with normal lung function. We can detect regional lung function impairments (especially in PRISm patients) in patients with FEV1/FVC ≥ 0.7, which may be significant for early COPD diagnosis.

## Supplementary Information

Supplementary Material 1.

## Data Availability

The datasets used and/or analyzed during the current study are available from the corresponding author on reasonable request.
