# Supplementary material for: Regional lung function assessment using electrical impedance tomography in COPD, PRISm, and normal spirometry subjects: insights into early diagnostic potential
Source: BMC Pulm Med. 2025 May 5;25:215. doi: 10.1186/s12890-025-03668-z (PMC12051352; doi:10.1186/s12890-025-03668-z)
Supplement: Supplementary file 1 — Supplementary Material 1. [file 12890_2025_3668_MOESM1_ESM.docx]

Supplementary method：

After ventilation-related data were collected via EIT, regional lung function was analyzed via customized software (Matlab 2013a, Mathwork, MA, US). After data reconstruction, a single EIT image typically contains 32×32 pixels. Data analysis is mainly as follows:

**Regional Lung Function Image Construction**

Firstly, determine the pulmonary regions (analysis areas) based on ventilation. Pixels with relative impedance changes (ΔZ) higher than 20% of the maximum ΔZ are considered pulmonary regions, and all subsequent calculations are confined to these areas. Within the pulmonary region pixels, calculate the difference between the maximum relative impedance value (Zmax) reached after forced full inhalation to total lung capacity and the minimum relative impedance value (Zmin) after forced exhalation to residual volume. This difference reflects the forced vital capacity (FVC_EIT_) of each pixel. By comparing the sum of all pixel FVC_EIT_ values with the FVC measured by spirometry, convert the arbitrary units (AU) of impedance to milliliters using the following formula:

$Z\left( in ml \right)=Z (in AU)\times\frac{FVC (in ml)}{{FVC}_{EIT} (in AU)}$

Another parameter determined by forced vital capacity is the forced expiratory volume in one second (FEV1_EIT_), which calculates the difference in relative ΔZ values at one second after forced exhalation and the lung capacity value at the start of the exhalation.

To describe the dispersion of fEIT images, we calculated the global inequality (GI) index and for each type of EIT. For FEV1/FVC_EIT_, we calculated the percentage of pixels with values less than 0.7 as a percentage of the total number of pixels in the pulmonary region. This parameter is expressed as “abnormal%.”

**Regional Lung Function Image Construction (Temporal Images):**

Temporal images characterize the regional time distribution of different expiratory volumes and their coefficient of variation to precisely reflect the time required for unit expiratory volume, objectively representing the explosive force and endurance of the respiratory muscles, as well as whether there is functional damage to the lungs.

**Time constant map:** For each pixel in the pulmonary region, the time constant is calculated by fitting the following exponential equation:

$Z\left( t \right)=Z_{0}\cdot e^{\frac{-t}{\tau}}+c$

where *Z*(*t*) is the relative impedance of the pixel in the pulmonary region at time *t*, Z_0_ is the impedance at the start of exhalation, *t* represents the time from the end of inhalation to the end of exhalation, *τ* is the regional time constant, and *c* represents the residual volume at the end of exhalation. Unlike previous calculations for each tidal breath, here the time constant is calculated based on FVC data.

Supplementary data processing

The study included only patients with complete data, whereas those with missing data were excluded.

Supplement tables and figures

FIGURE 1 EIT parameters after the bronchodilation test.


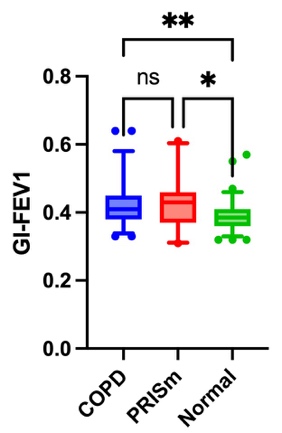

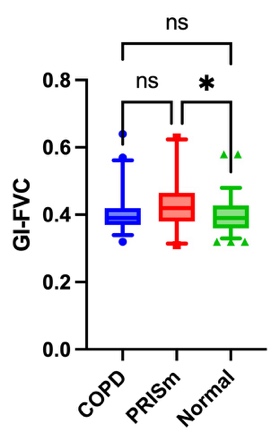

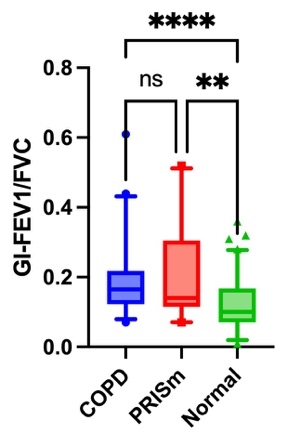

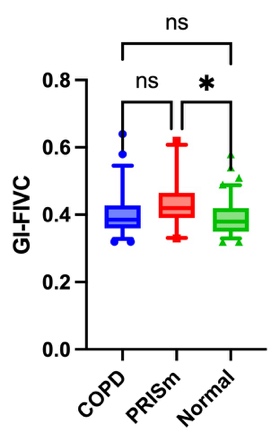


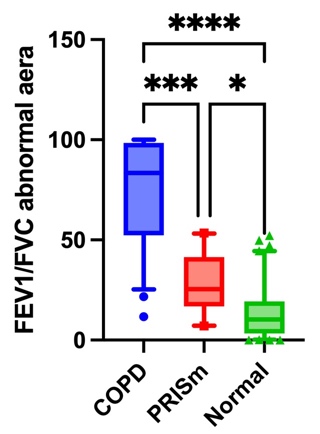

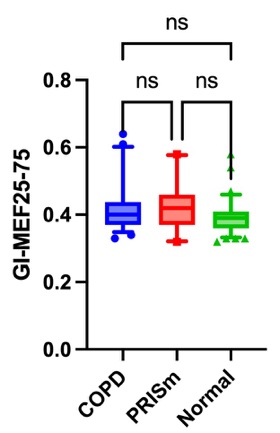

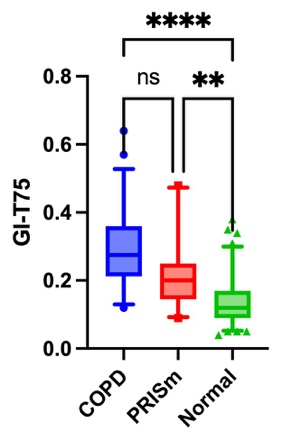

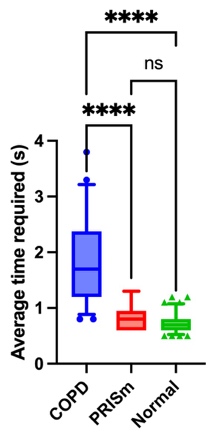


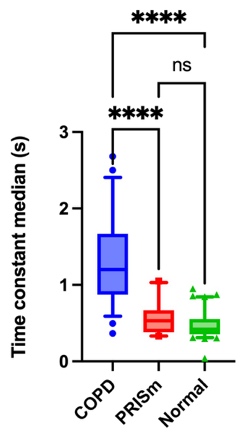

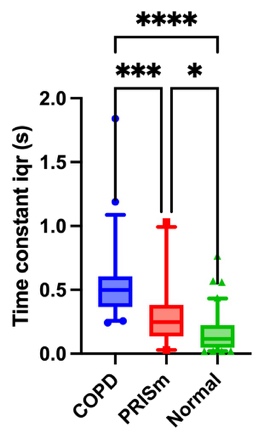

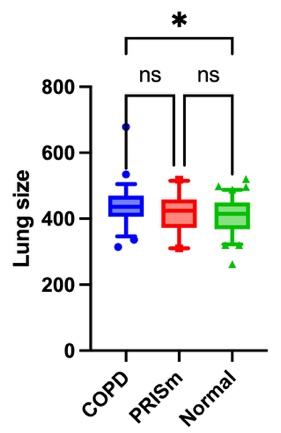

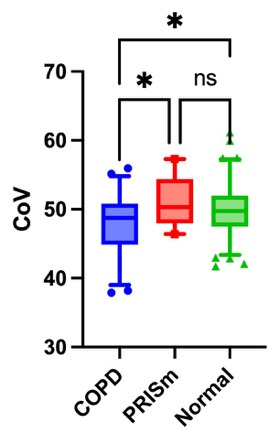


Global inhomogeneity (GI) of electrical impedance tomography derived regional pulmonary function measures of forced expiratory volume in 1 s (FEV1), forced vital capacity (FVC) , FEV1/FVC, forced inspiration vital capacity (FIVC), middle expiratory flow rate at 25% to 75% of FVC (MEF25-75), time required to exhale 75% of FVC (T-75) in COPD, PRISm, Normal patients; FEV1/FVC abormal aera: pixels with an fEIT FEV1/FVC ratio less than 0.7 of the total lung region pixels; CoV: center of ventilation; Averaged time required: the time required to exhale 75% of FVC; Time constant: as numerical statistics, the median (τmed) and interquartile range (τiqr) of regional time constants. Multiple comparison test are indicated by asterisks. *: p<0.05; **: p<0.01; ***: p<0.001; ****: p<0.0001.

FIGURE 2 ROC curves

1. **
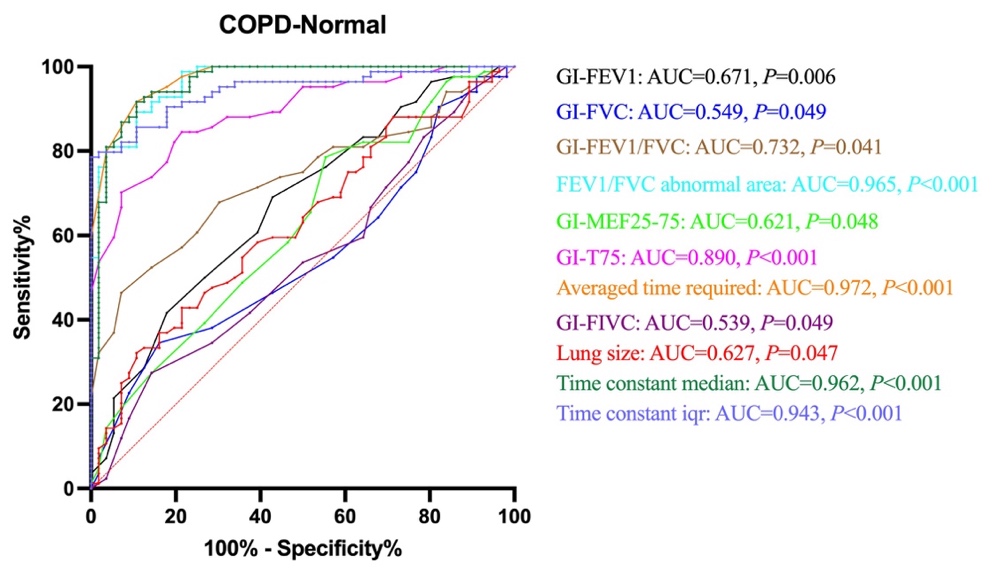
**
2.
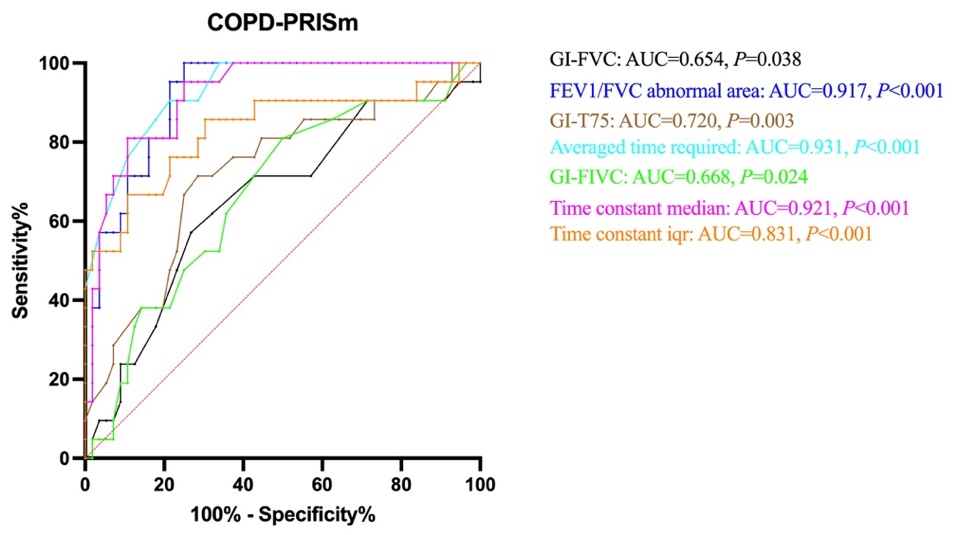


C)
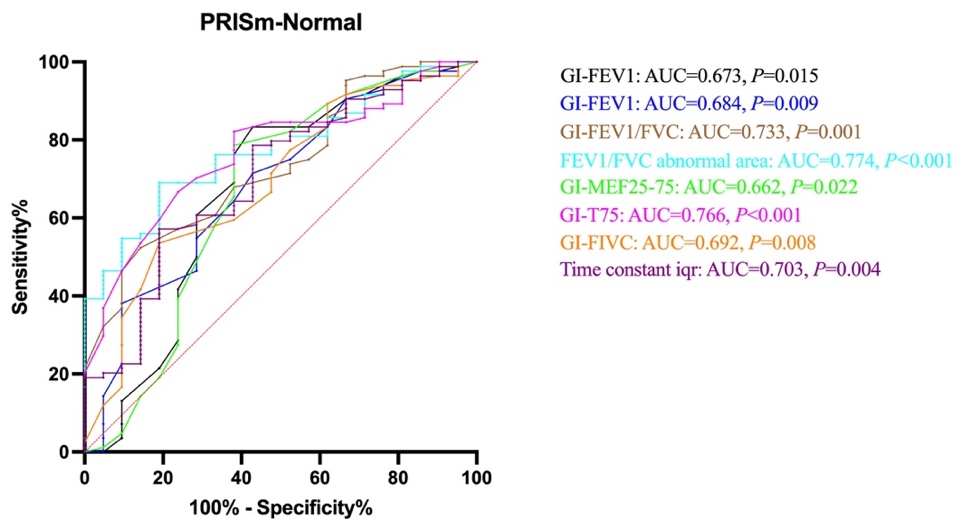


Receiver - operating characteristic (ROC) curves of EIT - related parameters for distinguishing among the three groups. The reference line and P - value are given in each graph. AUC represents the area under the curve. Figure A show the discrimination between the COPD group and the Normal group, figure B shows the discrimination between the COPD group and the PRISm group, and figure C shows the discrimination between the PRISm group and the Normal group.

TABLE 1 Change values in EIT parameters and pulmonary function testing (PFT) results after the bronchodilation test

| Variables | Normal (n=84) | COPD(n=56) | PRISm (n=21) | *P* |
| --- | --- | --- | --- | --- |
| GI FEV1  GI FVC  CoV  GI FEV1/FVC  FEV1/FVC abnormal area  GI-MEF25-75  GI-T75  Average time required  GI-FIVC  Lung Size  τmed  τiqr  FVC% pred  FEV1%pred  FEV1/FVC | 0.00 (-0.02-0.02)  0.00 (-0.02-0.03)  -0.06 (-0.89-0.68)  0.00 (-0.05-0. 03)  -2.62 (-9.60-4.29)  0.00 (-0.03-0.02)  -0.01 (-0.04-0.01)  0.00 (-0.10-0.00)  0.00 (-0.03-0.03)  10.00 (-6.00-29.00)  -0.02 (-0.08-0.01)  -0.01 (-0.07-0.01)  0.05 (-2.65-2.10)  2.10 (-0.30-4.35)  2.07 (0.75 -3.40) | -0.01 (-0.04-0.03)  0.00 (-0.02-0.03)  0.07 (-0.70-1.19)  -0.01 (-0.05-0.04)  -1.31 (-10.73-3.80)  -0.01 (-0.04-0.03)  0.05 (-0.06-0.06)  -0.10 (-0.30-0.10)  0.00 (-0.02-0.03)  7.00 (-14.00-29.50)  -0.11 (-0.30-0.03)  -0.03 (-0.20-0.10)  3.15 (0.70-7.48)  1.22 (-1.63-2.52)  3.15 (0.43-6.78) | 0.00 (-0.02-0.06)  0.02 (-0.02-0.04)  0.44 (-0.21-0.81)  0.01 (0.01-0.09)  -2.31 (-7.58-5.35)  0.00 (-0.02-0.06)  0.01 (-0.04-0.07)  -0.10 (-0.10-0.00)  0.02 (-0.03-0.05)  12.00 (0.00-32.00)  -0.01 (-0.06-0.12)  0.02 (-0.01-0.09)  1.30 (-0.50-6.00)  2.90 (0.40-6.10)  1.54 (0.31-4.35) | .603  .609  .269  .116  .932  .526  .178  .084  .363  .420  .047  .097  .000  .180  .042 |

EIT: electrical impedance tomography. PFT: pulmonary fucnction tests. GI: global inhomogeneity. FEV1: forced expiratory volume in 1 s; FVC: forced vital capacity. MEF: maximal expiratory flow. FIVC: forced inspiration vital capacity.

TABLE 2 Comparison of PFT and EIT between never-smokers and current/former smokers in FEV1/FVC <0.7

| Variables | Smoke (n=35) | No smoke (n=21) | *P* value |
| --- | --- | --- | --- |
| GI FEV1  GI FVC  CoV  GI FEV1/FVC  FEV1/FVC abnormal area  GI-MEF25-75  GI-T75  Average time required  GI-FIVC  Lung Size  τmed  τiqr  FVC% pred  FEV1%pred  FEV1/FVC | 0.42 (0.39-0.46)  0.39 (0.38-0.41)  48.43 (44.25-50.22)  0.18 (0.13-0.22)  83.61 (53.79-98.60)  0.40 (0.37-0.43)  0.26 (0.18-0.36)  1.70 (1.25-2.60)  0.38 (0.36-0.40)  446.68±59.86  1.20 (0.91-1.75)  0.47 (0.37-0.59)  82.10±16.32  61.78±20.20  57.1±11.00 | 0.38 (0.38-0.42)  0.40 (0.37-0.44)  49.25 (46.61-50.88)  0.16 (0.12-0.21)  80.00 (60.63-96.30)  0.40 (0.38-0.45)  0.30 (0.34-0.34)  1.50 (1.20-2.0)  0.40 (0.37-0.44)  419.23±44.02  1.20 (0.88-1.36)  0.53 (0.44-0.63)  82.77±18.27  61.19±15.16  58.66±7.62 | .321  .658  .370  .553  .611  .872  .275  .213  .269  .066  .446  .365  .912  .926  .919 |

TABLE 3 Comparison of PFT and EIT between never-smokers and current/former smokers in FEV1/FVC ≥0.7

| Variables | Smoke (n=36) | No smoke (n=69) | *P* value |
| --- | --- | --- | --- |
| GI FEV1  GI FVC  CoV  GI FEV1/FVC  FEV1/FVC abnormal area  GI-MEF25-75  GI-T75  Average time required  GI-FIVC  Lung Size  τmed  τiqr  FVC% pred  FEV1%pred  FEV1/FVC | 0.38 (0.37-0.43)  0.40 (0.35-0.43)  49.67±2.99  0.12 (0.09-0.17)  16.05 (8.16-34.33)  0.38 (0.35-0.42)  0.15 (0.11-0.21)  0.80 (0.70-0.90)  0.39 (0.36-0.43)  434.67±51.48  0.52 (0.41-0.63)  0.21 (0.09-0.34)  93.93±14.85  91.37±13.97  77.75±4.91 | 0.39 (0.37-0.42)  0.40 (0.36-0.43)  50.40±4.05  0.11 (0.07-0.20)  10.47 (3.58-21.71)  0.40(0.37-0.42)  0.12 (0.09-0.18)  0.70 (0.60-0.80)  0.39 (0.36-0.43)  398.78±50.63  0.40 (0.35-0.53)  0.11 (0.05-0.22)  94.78±12.83  93.64±13.36  82.07±6.10 | .187  .287  .345  .985  .049  .147  .084  .006  .692  .000  .005  .020  .760  .417  .000 |

EIT: electrical impedance tomography. PFT: pulmonary fucnction tests. GI: global inhomogeneity. FEV1: forced expiratory volume in 1 s; FVC: forced vital capacity. MEF: maximal expiratory flow. FIVC: forced inspiration vital capacity.
